# Case Report: Experience of a rare case of primary acute mast cell leukemia with FGFR1 gene rearrangement

**DOI:** 10.3389/fonc.2026.1830652

**Published:** 2026-05-07

**Authors:** Yalu Liu, Sheng Gao, Huizhen Du, Hong Ji, Zhao Li

**Affiliations:** 1 Department of Hematology, Qilu Hospital (Qingdao), Cheeloo College of Medicine, Shandong University, Qingdao, Shandong, China; 2 Department of Laboratory, Qilu Hospital (Qingdao), Cheeloo College of Medicine, Shandong University, Qingdao, Shandong, China; 3 Department of Pathology, Qilu Hospital (Qingdao), Cheeloo College of Medicine, Shandong University, Qingdao, Shandong, China

**Keywords:** case report, FGFR1, gene rearrangement, KIT mutation, mast cell leukemia

## Abstract

Mast cell leukemia represents the most malignant subtype of systemic mastocytosis, characterized by a dire clinical prognosis. Currently, no standard treatment regimen exists, with allogeneic hematopoietic stem cell transplantation being the sole option that may extend patient survival. Most mastocytosis patients exhibit mutations in the KIT gene. This paper presents a case of mast cell leukemia manifesting primarily as intractable shock and extensive bone destruction. Notably, the patient tested negative for KIT gene mutations but exhibited an FGFR1 gene rearrangement. Initial treatment with venetoclax and azacitidine proved ineffective, prompting a shift to second-line targeted therapy using an FGFR1 inhibitor. This case is the first to report FGFR1 gene rearrangement in a mast cell leukemia patient, offering deeper molecular insights into this highly aggressive disease.

## Introduction

Mast cell leukemia (MCL) represents an exceptionally rare and aggressive hematologic malignancy, constituting the most advanced subtype of systemic mastocytosis (SM). It is defined by the abnormal growth and aggregation of clonal mast cells (MCs) (1, 2). As the most aggressive and least common variant of SM, MCL comprises fewer than 1% of all diagnosed cases. MCL is diagnosed based on established criteria for SM (2–6). Unlike indolent SM, which typically follows a benign clinical course with a normal or near-normal life expectancy, MCL is marked by rapid proliferation of immature MCs, leading to severe organ dysfunction and significantly reduced survival (6–8).

The prognosis of MCL remains extremely poor, and a standardized first-line therapy is lacking. Although the KIT D816V variant represents the predominant genetic driver in SM, emerging evidence indicates that KIT D816V-negative status is linked to inferior treatment responses and shorter overall survival (9). Furthermore, MCL frequently harbors additional bone marrow-related gene mutations, including TET2, SRSF2, ASXL1, SF3B1, and RUNX1 (4, 10, 11). No standard care has been established for MCL. While treatments such as midostaurin, single-agent cladribine (2-CdA), or multi-agent acute myeloid leukemia (AML)-type induction regimens are employed, they rarely achieve durable long-term responses. At present, allogeneic hematopoietic stem cell transplantation remains the sole treatment option that may extend survival (12). Our research details a rare case of primary acute MCL with wild-type KIT, notably marked by a rearrangement of the fibroblast growth factor receptor 1 (FGFR1) gene, thereby offering new insights into potential therapeutic strategies.

## Case presentation

A 71-year-old male patient without a history of chronic illness presented with epigastric pain predominantly during fasting and nocturnal periods in early December 2024, without any obvious triggers. As time progressed, his symptoms worsened, manifesting as vomiting and constipation. Preliminary laboratory tests indicated the presence of leukocytosis, mild anemia, and thrombocytopenia. Oral moxifloxacin therapy was initiated for a suspected infection, but the therapeutic response was suboptimal. On January 13, 2025, the patient exhibited symptoms including fever and cough. These symptoms were temporarily mitigated following self-administered antipyretics. On January 23, 2025, the patient sought further evaluation at our hospital due to worsened respiratory symptoms.

The complete blood count (CBC) revealed leukocytosis, thrombocytopenia, and anemia. The specific initial blood counts are presented in **Supplementary Table 1**. A computed tomography (CT) scan identified multiple osteolytic lesions in bilateral ribs, sternum, and vertebral bodies. Subsequent positron emission tomography-CT (PET-CT) indicated diffusely heterogeneous bone density with diffusely increased FDG metabolism (SUVmax 8.90), alongside focal hypermetabolic lesions (**Figure 1**). Additionally, PET-CT detected enlarged lymph nodes in the hepatotactic space and retroperitoneum, which exhibited mild metabolic activity (SUVmax 1.86).

**Figure 1:**
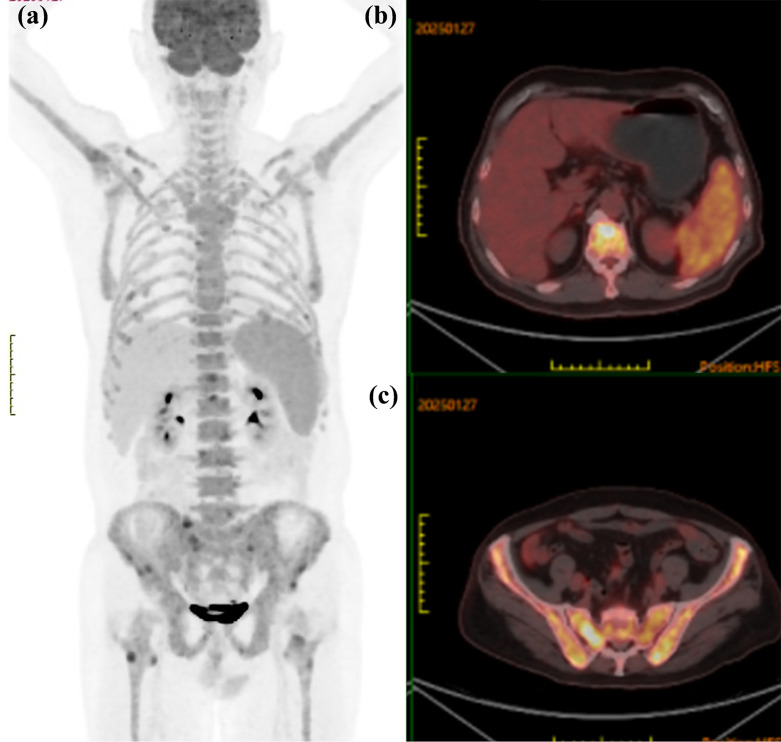
Pretreatment FDG PET-CT images of the patient. **(a)** maximum intensity projection PET image of the whole body. **(b)** axial fused PET-CT image demonstrating splenomegaly with diffusely increased FDG uptake and patchy low-density foci within the spleen (SUVmax, 5.02). **(c)** axial CT image showing heterogeneous bone density with patchy low-density areas and focally increased FDG uptake (SUVmax, 8.90).

Prior to a definitive diagnosis, the patient suddenly developed hypotensive shock, with his blood pressure falling to 81/48 mmHg. This critical event was accompanied by significantly elevated D-dimer, progressive thrombocytopenia, and a 10-fold elevation in lactate dehydrogenase (LDH) levels. Collectively, these clinical manifestations and laboratory findings indicated a state of disseminated intravascular coagulation (DIC). Following transfer to the intensive care unit (ICU), the patient was hemodynamically stabilized through fluid resuscitation, norepinephrine infusion for blood pressure support, and antimicrobial therapy.

### Diagnostic assessment

To confirm the diagnosis, a bone marrow aspiration was performed. Morphological examination of the sample revealed 17% MCs and 57% reactive plasma cells. Flow cytometry further identified 17.54% abnormal MCs and a distinct population of cells (“pink-staining cell cluster”), which accounted for 63.5% of nucleated cells (**Figure 2a**). Immunophenotypically, this population exhibited strong CD33 expression and weak CD11b expression, while being negative for CD13, CD117, CD34, and CD203c, with co-expression of CD2. Notably, flow cytometry failed to determine the cellular origin of these “pink-staining cell cluster”.

**Figure 2:**
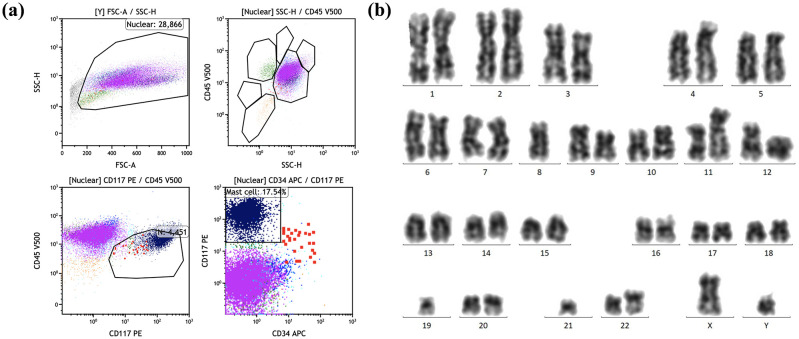
Bone marrow flow cytometry and chromosome karyotype. **(a)** bone marrow flow cytometry. **(b)** among the 20 cells analyzed, 20 were der(21;22)(q10;q10)del(22)(q11q12), and 12 of them were accompanied by dic(8;11).(p12; q12), r (12) (p13q24), - 19, visible cloned abnormal dic (8, 11), r (12), 19, der (21, 22) del (22 q).

Immunohistochemical (IHC) tests were performed on the bone marrow specimen to further characterize the “pink-staining cell cluster”. These abnormal cells were positive for CD25, CD117, CD2, and tryptase, consistent with abnormal MCs (**Figure 3**).

**Figure 3:**
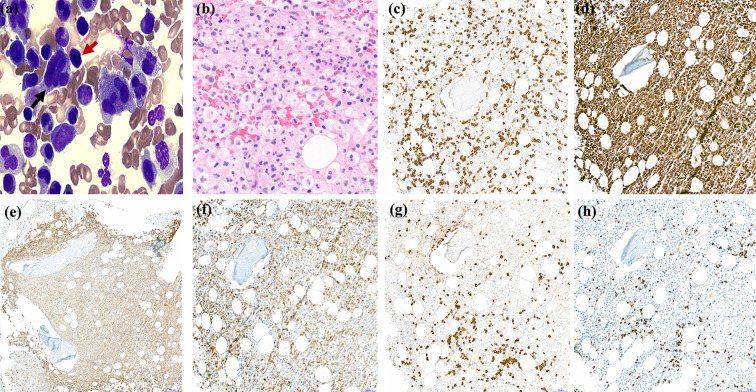
Morphological analysis and immunohistochemistry of the bone marrow biopsy. **(a)** representative image showing typical (red arrow) and atypical (black arrow) mast cells. **(b)** hematoxylin-eosin (H&E) staining of the bone marrow biopsy (original magnification, ×40). Immunohistochemical staining shows that the abnormal cells are positive for CD117 **(c)**, CD68 **(d)**, CD25 **(e)**, and CD2 **(f)**, and negative for MPO (myeloperoxidase) **(g)** and lysozyme (LYSO) **(h)**. [original magnification, ×20 for panels **(c–h)**].

According to the NCCN Guidelines for Systemic Mastocytosis, this patient was diagnosed with MCL. Sequencing analysis using the Illumina high-throughput sequencing platform revealed a wild-type KIT gene. Unexpectedly, high-throughput sequencing also identified mutations in SETD2 and GNB1, along with multiple copy number variations (**Supplementary Table 2**). Moreover, fluorescence *in situ* hybridization (FISH) using a three-color break-apart FGFR1/D8Z2 probe demonstrated positivity for FGFR1 gene rearrangement (**Figure 4**). Cytogenetic analysis showed a complex karyotype, indicating a poor clinical prognosis (**Figure 2b**). Based on the NCCN guideline, this patient was classified as high-risk, with a mutation-adjusted risk score of 3.

**Figure 4:**
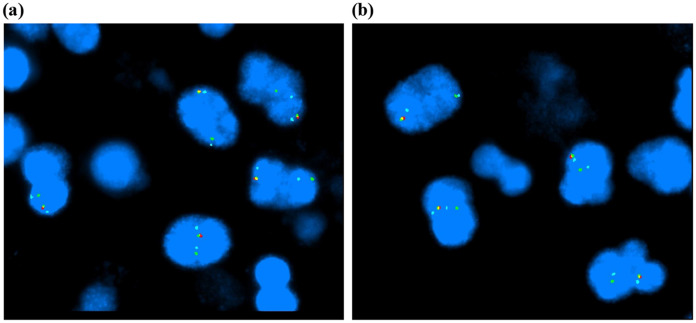
FISH analysis of BM cells using an FGFR1 break-apart DNA probe, revealing an FGFR1 rearrangement (green, 5’FGFR1; red, 3’FGFR1). **(a)** normal control. **(b)** FGFR1 rearrangement-positive.

### Management and disease progression

Given the absence of a standard first-line therapy for MCL, patients are advised to enroll in an appropriate clinical trial or to consider AML-based therapy incorporating cladribine or midostaurin. Shortly before a definitive diagnosis was established, the patient suddenly developed shock of unclear etiology. The presence of fever, elevated procalcitonin and high-sensitivity C-reactive protein, along with a favorable response to antibiotic therapy, pointed toward septic shock. Concurrently, leukocytosis, elevated lactate dehydrogenase, and metabolic acidosis were observed, all of which are consistent with mast cell activation. The fact that the patient stabilized with antibiotic therapy alone suggests that septic shock was the dominant mechanism. However, given the underlying mast cell leukemia, it is likely that infection-triggered mast cell activation contributed to the severity of the shock. In light of his poor clinical condition, he was deemed unfit. The first-line treatment followed the established approach for older, unfit AML cases. Venetoclax combined with azacitidine (azacitidine 75 mg/m² × 7 days) was administered.

After the first treatment cycle, bone marrow aspiration was repeated, revealing that MCs constituted 83% of the marrow cellularity, with peripheral blood MCs accounting for 33%. The treatment response of this patient was evaluated as non-remission (NR) due to a significant increase in marrow MC infiltration. For the second treatment cycle, the FGFR1 inhibitor combined with cladribine and low-dose cytarabine was administered. The patient’s treatment process is summarized in **Supplementary Figure 1**. The patient developed a pulmonary infection during the course of chemotherapy-induced myelosuppression, which ultimately led to respiratory failure and death. The interval between diagnosis and death was approximately 60 days.

## Discussion

This report presents an exceptionally rare case of MCL, characterized by elevated leukocyte counts and multiple bone destructions. Remarkably, the present case exhibited a negative KIT gene mutation and a positive FGFR1 gene rearrangement, offering novel insights into the diagnosis and treatment of MCL.

In 2016, the World Health Organization (WHO) reclassified mastocytosis as a distinct disease entity marked by the abnormal growth and aggregation of clonal MCs in various organs (13, 14). Both the WHO and International Consensus Classification (ICC) standards identify MCL as the most advanced and lethal stage of SM (15). However, due to the rarity of MCL, the available information is predominantly derived from single case reports, small series, and registry studies, thereby limiting the depth of knowledge and making it challenging to conduct robust clinical trials. The current discussion focuses on combining the molecular characteristics and treatment plan of this case to explore challenges in its diagnosis, its association with known prognostic factors, and targeted therapy.

The clinical manifestations of MCL are highly non-specific. MCL is thus often misdiagnosed as an infection or systemic illness, leading to diagnostic delay. In patients presenting with unexplained cytopenias, refractory hypotension, and multiple bone destruction, MCL should be considered even in the absence of classic symptoms for degranulation of MCs. Infiltrates of MCs can be mistaken for other myeloid proliferations, and the use of a critical MC IHC panel (tryptase, CD117, CD25, CD2) is essential for accurate diagnosis. In this particular case, immunohistochemistry of the bone marrow specimen showed that 74% of the infiltrating abnormal cells expressed CD25, CD117, and CD2. Furthermore, these cells were also positive for trypsin-like protease and confirmed as MCs, thereby meeting the diagnostic criteria for MCL.Furthermore, MCL harboring an FGFR1 rearrangement should be differentiated from MCL with an associated hematological neoplasm (MCL-AHN). Although both entities may present with FGFR1 rearrangement and proliferation of MCs, the diagnosis of MCL-AHN requires evidence of a distinct associated myeloid neoplasm. The presence of AHN represents a significant feature in a considerable number of individuals with MCL. Studies have reported that MCL-AHN occurs in 34% to 71% of cases. In this particular case, bone marrow morphology showed no evidence of dyserythropoiesis, ringed sideroblasts, multinuclearity, or myeloid blasts. Therefore, the possibility of MCL-AHN was ruled out, and a diagnosis of primary acute MCL was confirmed. This finding underscores the critical role of bone marrow examination in the diagnostic process.

The prognosis for MCL is exceptionally dismal, with an average survival rate of only about six months. MCL is frequently linked to somatic gain-of-function point mutations in the KIT gene. The KIT D816V mutation is the dominant variant in SM, with a detection rate ranging from 70% to 90% among patients. Other reported KIT variants are V560G, D815K, D816Y, insVI815-816, D816F, D816H, and D820G (16). The KIT receptor is a transmembrane protein that elicits diverse cellular responses depending on the cell type. These responses involve pathways such as PI3K-Akt, Src kinases, Ras-Erk, phospholipase C, MAPK, and JAK-STAT (17–19). Mutations in the KIT receptor tyrosine kinase are pivotal in the development of MCL by driving receptor activation, which fosters uncontrolled proliferation of MC and enhances cell survival by impairing apoptosis. However, approximately 16% of MCL cases lack KIT mutations, and the pathogenesis is unclear. Wild-type KIT is present in the cytosol and cell membrane. Previous research confirms that wild-type KIT requires SCF binding for phosphorylation, whereas KIT D816V can auto-phosphorylate independently (20). Patients without the KIT D816V mutation exhibit a poorer treatment response and lower overall survival compared to those with this mutation (21). ECNM registry data have also confirmed that negative KIT D816V is correlated with worse overall survival (HR = 0.33; P = .04) (6). For patients with wild-type KIT, genetic analysis can identify alternative driver mutations. These findings may have diagnostic, prognostic, and potential therapeutic implications. As demonstrated in the present case, genetic testing revealed an FGFR1 rearrangement, which not only confirmed the diagnosis but also suggested a potential therapeutic target, although its clinical benefit could not be fully evaluated due to unforeseen complications. Therefore, we recommend that genetic testing (including next-generation sequencing and FISH) be routinely performed in patients with MCL, especially in those with wild-type KIT, to identify actionable alterations and optimize prognostic stratification.

The most notable molecular characteristic in the present case is the FGFR1 gene rearrangement. The present case appears to be the first report of MCL linked to FGFR1 rearrangement, to date. This finding contributes new insights to the molecular spectrum of this highly aggressive disease. FGFR1 gene rearrangement is a rare driver mutation primarily associated with 8p11 myeloproliferative syndrome (EMS) (22–26). However, reports of SM caused by FGFR1 gene rearrangement are extremely rare. Chang et al. report the first case of FGFR1 translocation in a patient with concurrent lymphoblastic lymphoma, myeloproliferative neoplasm, and SM. The presence of CD25-expressing MCs was noted in the lymph node biopsy of this patient, and the sequencing revealed t(8,13)(p11;q12) involving FGFR1 and ZMYM2 (27). Loscocco et al. also report a case of myeloid/lymphoid neoplasm with SM-associated ZMYM2::FGFR1 rearrangement, which rapidly transformed into AML (28). Additionally, the most prevalent FGFR1 translocation partner gene is ZNF198 on chromosome 13q12 (29). Chao et al. previously reported an extremely rare case of acute eosinophilic leukemia with a positive ZNF198-FGFR1 rearrangement (30). However, our study is limited by the absence of any partner genes linked to FGFR1.

Pemigatinib is a highly selective FGFR1–3 inhibitor that exerts antitumor effects by targeting and inhibiting the FGFR signaling pathway (31). Based on the phase II FIGHT-203 study involving 47 patients, pemigatinib demonstrates marked efficacy in FGFR1-rearranged myeloid/lymphoid neoplasms (MLN-FGFR1), achieving a complete cytogenetic response rate of 73% (32). These findings confirm that targeted inhibition of FGFR1 fusions can induce deep and durable hematologic and cytogenetic remissions. Given the marked efficacy of pemigatinib in MLN-FGFR1, the inhibition of FGFR1 may constitute a plausible treatment approach for MCL characterized by FGFR1 activation or aberrant FGFR signaling. However, data regarding the use of pemigatinib in MCL remain extremely limited. To date, reports of its application in MCL are restricted to isolated case descriptions. In the present case, after the failure of first-line therapy with venetoclax combined with azacitidine, the patient received second-line treatment with pemigatinib and cladribine chemotherapy. Unfortunately, the patient ultimately succumbed to respiratory failure due to the development of severe pneumonia during the chemotherapy-induced myelosuppression phase before the therapeutic effects of pemigatinib could be evaluated. The duration between diagnosis and death was two months. A review of the patient’s medical history revealed that the targeted agent, pemigatinib, was administered only after the first-line therapy failed due to economic reasons. By that time, the MCL remained refractory, prolonged agranulocytosis had developed, and severe infectious pneumonia ensued. Consequently, the targeted agent could not demonstrate potential benefit, and the patient died of a pulmonary infection. We contend that venetoclax plus azacytidine has limited efficacy for patients with MCL. Crucially, earlier administration of targeted agents may offer a realistic opportunity to improve prognosis. It should be acknowledged that, despite the rarity of FGFR1-positive MCL, this single case report provides only observational findings. The conclusions presented here cannot be generalized without adequate validation and integration with data from larger patient cohorts.

According to the recommendations, this patient received a mutation-adjusted risk score of three, indicating a high-risk classification. The patient did not have mutations in ASXL1, RUNX1, or NRAS genes, but had mutations in SETD2 and GNB1 genes with frequencies exceeding 30% each. Mutations p.E252* and p.R1708Tfs*4 were identified in the SETD2 gene. Both are truncating mutations that may lead to abnormalities in the encoded protein (33, 34). The SETD2 gene encodes histone H3K36 methyltransferase. It functions as a tumor suppressor gene associated with various chromosomal aberrations observed in AML. A p.G116V mutation was detected in the GNB1 gene. This is the first reported mutation of this gene. The GNB1 mutation is an upstream mutant gene of BRAF in the RAS-BRAF-MAPK-ERK pathway, suggesting an association with a poor prognosis (35–37). Additionally, chromosomal karyotype analysis revealed that the patient had a complex karyotype, indicating a poor clinical prognosis.

This case study underscores the pivotal role of comprehensive genetic testing, including FGFR1 analysis, in MCL patients who do not respond to standard treatments, especially those with KIT wild-type mutations. In the event of an FGFR1 rearrangement, it is recommended that FGFR inhibitors be incorporated into the treatment regimen in a timely manner, rather than continuing to use alternative chemotherapy protocols. Nevertheless, the efficacy of targeted therapies in patients with MCL should be validated through further clinical studies.

## Data Availability

The original contributions presented in the study are included in the article/**Supplementary Material**. Further inquiries can be directed to the corresponding authors.
