# Supplementary figures and images for: Case Report: Experience of a rare case of primary acute mast cell leukemia with FGFR1 gene rearrangement
Source: Front Oncol. 2026 May 7;16:1830652. doi: 10.3389/fonc.2026.1830652 (PMC13189944; doi:10.3389/fonc.2026.1830652)

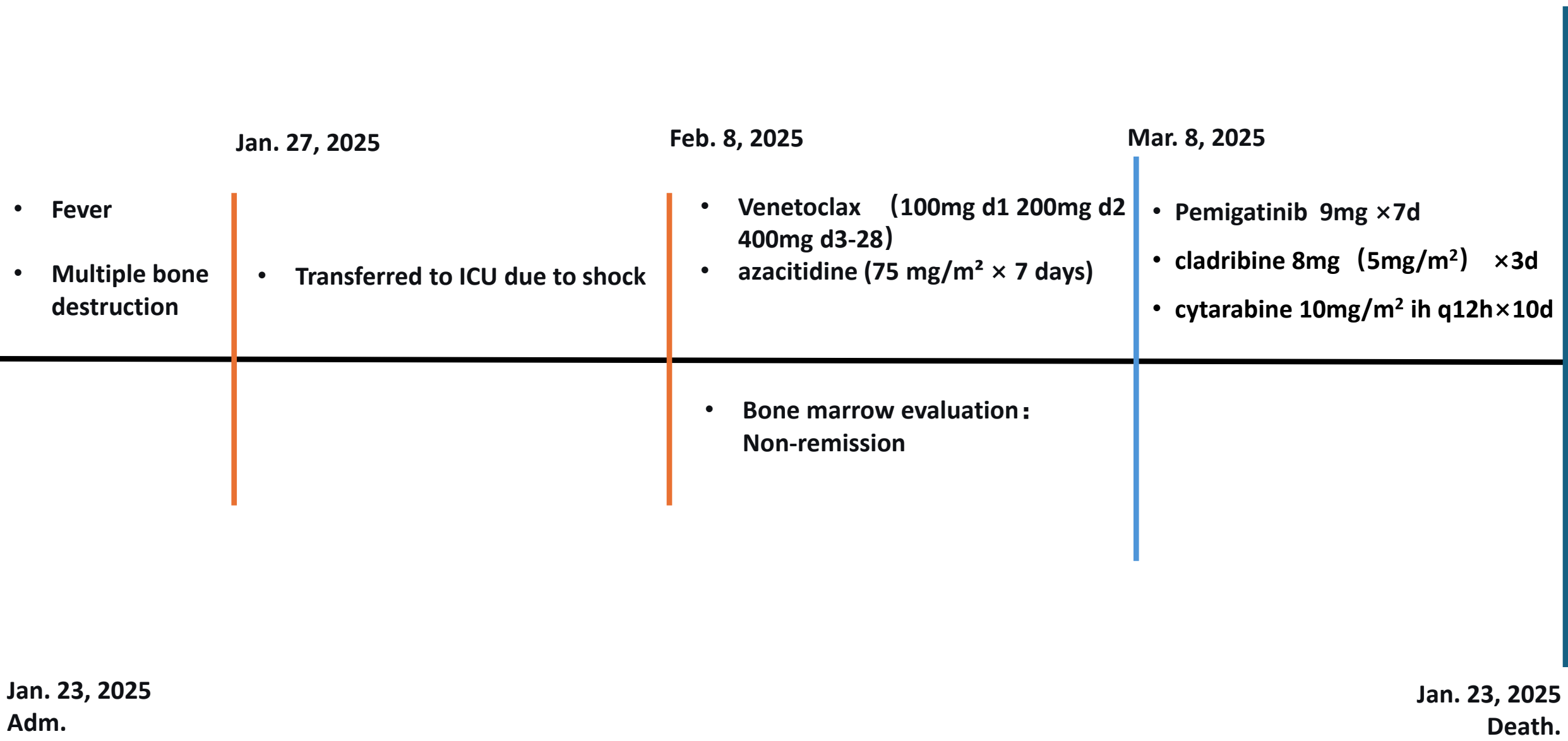

Supplement: Supplementary file 1 [file DataSheet1.pdf]
