# Supplementary material for: Case Report: Experience of a rare case of primary acute mast cell leukemia with FGFR1 gene rearrangement
Source: Front Oncol. 2026 May 7;16:1830652. doi: 10.3389/fonc.2026.1830652 (PMC13189944; doi:10.3389/fonc.2026.1830652)
Supplement: Supplementary file 2 [file Table1.docx]

**Supplementary Table 1. The patient's complete blood count at initial presentation**

| **Parameter** | **Result** | **Reference range** |
| --- | --- | --- |
| White blood cell count (×10^9/L) | 41.73 | 3.5–9.5 |
| Absolute neutrophil count (×10^9/L) | 16.77 | 1.8–6.3 |
| Absolute monocyte count (×10^9/L) | 16.07 | 0.1–0.6 |
| Absolute lymphocyte count (×10^9/L) | 8.26 | 1.1–3.2 |
| Absolute eosinophil count (×10^9/L) | 0.4 | 0.02–0.52 |
| Absolute basophil count (×10^9/L) | 0.23 | 0–0.06 |
| Hemoglobin (g/L) | 109 | 130-175 |
| Red blood cell count (×10^12/L) | 3.6 | 4.3-5.8 |
| Mean corpuscular volume (fL) | 89.2 | 82–100 |
| Platelet count (×10^9/L) | 92 | 125–350 |
