# Supplementary material for: Case Report: Experience of a rare case of primary acute mast cell leukemia with FGFR1 gene rearrangement
Source: Front Oncol. 2026 May 7;16:1830652. doi: 10.3389/fonc.2026.1830652 (PMC13189944; doi:10.3389/fonc.2026.1830652)
Supplement: Supplementary file 3 [file Table2.docx]

**Supplementary Table 2. Mutated genes detected by whole-exome sequencing**

| **Mutated Gene** | **Chromosome** | **Transcript ID** | **Variant Location** | **Nucleotide Change** | **Amino Acid Change** | **Variant Allele Frequency** | **Read Depth** |
| --- | --- | --- | --- | --- | --- | --- | --- |
| SETD2 | 3p21.31 | NM_014159 | exon21 | c.7558G>T | p.E2520* | 0.3627 | 1748X |
| SETD2 | 3p21.31 | NM_014159 | exon9 | c.5121dupA | p.R1708Tfs*4 | 35.85% | 2056X |
| GNB1 | 1p36.33 | NM_002074 | exon7 | c.347G>T | p.G116V | 51.87% | 3339X |
